# Interfacing poly(*p*-anisidine) with photosystem I for the fabrication of photoactive composite films†

**DOI:** 10.1039/d3na00977g

**Published:** 2023-12-22

**Authors:** Marc A. Nabhan, Allison V. Cordova-Huaman, David E. Cliffel, G. Kane Jennings

**Affiliations:** a Department of Chemical and Biomolecular Engineering, Vanderbilt University Nashville Tennessee 37235-1604 USA kane.g.jennings@vanderbilt.edu; b Department of Chemistry, Vanderbilt University Nashville Tennessee 37235-1822 USA

## Abstract

Photosystem I (PSI) is an intrinsically photoactive multi-subunit protein that is found in higher order photosynthetic organisms. PSI is a promising candidate for renewable biohybrid energy applications due to its abundance in nature and its high quantum yield. To utilize PSI's light-responsive properties and to overcome its innate electrically insulating nature, the protein can be paired with a biologically compatible conducting polymer that carries charge at appropriate energy levels, allowing excited PSI electrons to travel within a composite network upon light excitation. Here, a substituted aniline, 4-methoxy-aniline (*para*-anisidine), is chemically oxidized to synthesize poly(*p*-anisidine) (PPA) and is interfaced with PSI for the fabrication of PSI–PPA composite films by drop casting. The resulting PPA polymer is characterized in terms of its structure, composition, thermal decomposition, spectroscopic response, morphology, and conductivity. Combining PPA with PSI yields composite films that exhibit photocurrent densities on the order of several μA cm^−2^ when tested with appropriate mediators in a 3-electrode setup. The composite films also display increased photocurrent output when compared to single-component films of the protein or PPA alone to reveal a synergistic combination of the film components. Tuning film thickness and PSI loading within the PSI–PPA films yields optimal photocurrents for the described system, with ∼2 wt% PSI and intermediate film thicknesses generating the highest photocurrents. More broadly, dilute PSI concentrations show significant importance in achieving high photocurrents in PSI–polymer films.

## Abstract 2

The trans-membrane protein photosystem I is incorporated with the semiconducting polymer poly(*p*-anisidine) for the fabrication of biohybrid photoactive films.

## Graphical abstract

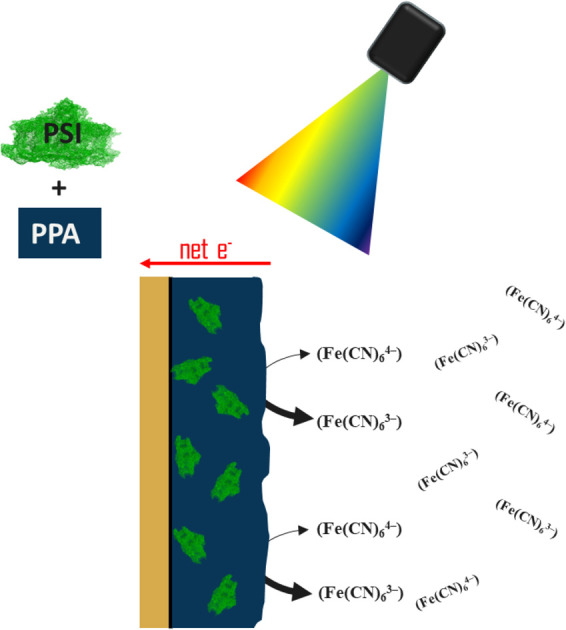

## Introduction

Acute climate change and depletion of resources are driving the development of alternative energy sources towards more sustainable and environmentally friendly technologies.^1^ While a plethora of the current renewable energy sources, namely in solar-energy conversion applications, have shown great promise in efficiencies and long-term stability, most of these technologies rely on rare elements, other expensive materials, laborious manufacture, and/or energy-intensive processes.^2^ Up until 2010, the photovoltaic (PV) industry was a net consumer of electricity, rather than a net producer, and is just now becoming a measurable producer.^3^ The shortcomings of these current technologies have motivated the exploration of biohybrid applications that make use of nature's pre-existing solar energy conversion mechanisms. The photosystem I (PSI) protein is a prime example of a naturally abundant bionanoparticle, and it has gained a growing interest in the last few decades due to its high quantum yield and significant photoactivity as one of the two biomacromolecular drivers of photosynthesis.^4^

PSI is found in the thylakoid membrane of chloroplasts where it plays a vital role in the electron transport chain (ETC) of photosynthetic organisms. In its natural environment, this multi-subunit membrane protein complex ensures electron transfer from plastocyanin/cytochrome c6 on the lumenal side of the membrane to ferredoxin/flavodoxin at the stromal side of the membrane. The redox potential of the protein is dictated by the energy level of its active sites with an iron-sulfur cluster, F_B_, serving as the terminal electron acceptor in the protein's ETC at −580 mV *vs.* NHE, the highest reducing potential in nature, and a P_700_ electron donor residing at +495 mV *vs.* NHE, summing to nearly 1.1 V of redox potential across PSI's membrane.^5^ Moving from P_700_ to F_B_, electrons hop rapidly across a series of co-factors in PSI's ETC chain, yielding an electron transfer turnover rate of 50 e^−^ s^−1^ per PSI *in vivo*.^6–8^

PSI has been integrated in a variety of energy conversion technologies in both wet and dry systems.^9^ The wide spectrum of PSI applications includes photoelectrochemical cells, dye-sensitized solar cells, solid-state devices, and photo-sensing applications.^4,10–17^ To make use of PSI's photoactivity, but to also compensate for its insulating nature, the protein has been integrated with a wide array of conducting polymers and electron transfer mediators^11,18^ such as Os-rich redox polymers,^19^ poly(benzyl viologen),^20^ PEDOT:PSS,^5,21^ polyaniline,^22^ and polyviologen.^23^ To efficiently interact with PSI's active sites, the polymer transfers holes or electrons corresponding to favorable energy levels to those of P_700_ or F_B_. Aniline undergoes redox reactions at +0.63 V *vs.* NHE, and many of its substituents follow suit with oxidation potentials ranging between +0.5 and +0.8 V.^24^ One aniline substituent, *para*-methoxyaniline (*p*-anisidine), stands out in having an oxidation potential of + 0.393 V *vs.* NHE, making it a potential candidate for electron/hole exchange with the P_700_ site of PSI (0.495 V *vs.* NHE).^25^ Aniline and many of its derivatives polymerize by oxidative polymerization through the amine group and the vacant *para* position as in the case of polyaniline (PANI) and poly(*o*-anisidine) (POA).^26–29^ Poly(*p*-anisidine) (PPA) is unique in that the *para* position is already occupied by the methoxy group, resulting in a less predictable polymer structure with both the *meta* and *ortho* positions being vacant. Our successful attempt at electropolymerizing *p*-anisidine into poly(*p*-anisidine), as shown in Fig. S1,† aligns with the previous literature where a conductive solid film can successfully be deposited onto a gold surface.^25^ The conductive nature of PPA, its energy alignment with the P_700_ site, and its potential for PSI biocompatibility, are all contributing motivators for the mixing of PSI proteins with the PPA polymer in the exploration of photoactive films.

PPA has mainly been reported to be synthesized *via* electropolymerization and is commonly employed as a component of a nanocomposite in the process.^30,31^ The electrodeposited PPA film is challenging to collect in solution, pushing us towards other synthesis procedures for controlled PSI–PPA mixing.^31^ Chemical oxidation is another common synthesis technique that has been widely used to yield conducting polymers in solution. PPA has been chemically polymerized in the presence of mixed components and introduced into nanocomposites by incorporating the polymer with various moieties such as titanium IV oxide and PEDOT:PSS during the polymerization process.^32,33^ The efforts of Rathidevi *et al.* and Rodrigues de Oliveira *et al.* are noteworthy, as they focused on the pristine polymer's oxidative chemical synthesis and characterization.^34,35^ While Rathidevi *et al.* mainly targeted a PPA–ZnO nanocomposite, they reported multiple characterization techniques for the polymer itself, whereas Rodrigues de Oliveira *et al.* examined the polymer's morphology, conductivity, and crystalline structure by considering the effect of varying concentrations of oxidant (ammonium persulfate) and dopant (HCl) on the resulting crystalline structure of PPA *via* chemical oxidation of *p*-anisidine. Rodrigues de Oliveira *et al.* reported that the chemical synthesis yielded a mixture of polymer structures, with varying proportions based on the synthesis conditions, where the C–N bonds that form during polymerization occur at the carbon either in the *ortho* or in the *meta* position of the repeating structure. They also report a modest conductivity for PPA ranging between 1.00 × 10^−9^ S cm^−1^ and 3.90 × 10^−14^ S cm^−1^, with a morphology described to bear globular and rod-like structures, as observed in their SEM images.

**Scheme 1:**
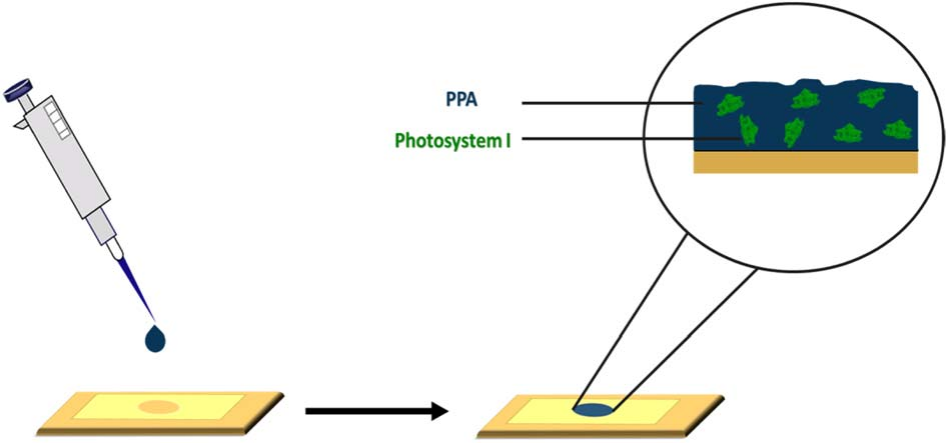
Drop casting deposition technique on a gold substrate mounted with an electrochemical mask used in PSI–PPA film fabrication.

Our aim in this study is first to chemically oxidize *p*-anisidine monomers into PPA in the absence of applied potential, and to compare the resulting polymer to those in literature. Second, we interface PPA with PSI for the fabrication of photoactive composite films. In the preparation of these composite films, we drop cast mixtures of PPA and PSI at various solution concentrations and ratios, as shown in Scheme 1, and we examine the effects of PSI loading in the film on photoelectrochemical properties. The resulting composite films are on the order of microns in thickness, are straightforward to fabricate, and exhibit higher photocurrents than that of either pure component. Our work here is unique in that we interface chemically polymerized PPA with a naturally abundant protein to fabricate photoactive films for photoelectrochemical biohybrid applications. We demonstrate synergistic photoelectrochemical effects between PPA and the PSI protein while characterizing the PPA polymer resulting from the described synthesis procedure and conditions.

## Experimental

### Materials and methods

#### Materials

PSI was extracted from organic baby spinach purchased at a local grocery store. Triton X-100 surfactant and 99% *p*-anisidine (4-methoxyaniline) were purchased from Millipore-Sigma. Silicon (100) wafers were purchased from WRS materials. Chromium-coated tungsten rods were purchased from R.D. Mathis. Gold shot (99.99% purity) was obtained from J&J Materials. Spectra/Por dialysis membrane tubing (6000 to 8000 Da MWCO) was obtained from Repligen. DMSO-*d*_6_ (99.5%) was purchased from Thermo Fisher Scientific.

#### PSI extraction

PSI was extracted from organic baby spinach following a previously described procedure.^36^ In short, spinach was de-veined, macerated, filtered, and then centrifuged at 8000*g*. The supernatant was then stabilized in aqueous solution with the help of Triton X-100 surfactant, before undergoing a second centrifugation at 20 000*g*. The resulting solution was then filtered through a hydroxyapatite column to exclude remaining impurities. The protein was dialyzed in a 1 : 4000 volume ratio of deionized water for 24 h using a 6–8 kDa MWCO dialysis tubing to remove salts and surfactant. A final concentration of 0.6 mg mL^−1^ for the extracted PSI solution was determined by measuring the thickness of a film drop cast from a known volume and deposited on a known surface area, assuming a protein density of 1.3 g cm^−3^.^37^

#### Preparation of gold substrates

The gold substrates were prepared following a previously described method.^38^ Silicon wafers were first washed with deionized water and ethanol, and then dried in a nitrogen stream. 10 nm of chromium (100 Å) and 125 nm of gold (1250 Å) were subsequently evaporated onto the processed silicon wafers at an evaporation rate of 2 Å s^−1^ or less. The evaporation was performed in a diffusion pumped chamber at a base pressure of 4 × 10^−6^ Torr. The wafers were then cut into 1.5 cm × 2.5 cm samples after evaporation. Prior to film deposition, gold substrates were rinsed with deionized water and then ethanol and then dried in a nitrogen stream.

#### Oxidative chemical polymerization

PPA was synthesized following modified polymerization procedures used to chemically polymerize aniline and *o*-anisidine.^27,39^*p*-Anisidine (0.02 mol) was dissolved in 200 mL of 1 M HCl (aq), and the monomer solution was maintained at −1 °C in an ethanol/water ice bath. The oxidant, ammonium persulfate ((NH_4_)_2_S_2_O_8_), was dissolved by adding 0.003 mol to 80 mL of 1 M HCl (aq). The polymerization solution was obtained by introducing dropwise the entire 0.04 M (NH_4_)_2_S_2_O_8_ oxidant solution into the 0.1 M *p*-anisidine solution, both initially at 1 M HCl, under vigorous stirring. The mixture was maintained at −1 °C for 2 h, resulting in the formation of a dark blue-green precipitate. The PPA solution was then dialyzed in a 1 : 2000 volume ratio of deionized water for 24 h using a 6–8 kDa MWCO dialysis tube to remove remaining salts and unreacted monomer. The conversion rate was determined at ∼80% where 8.8 mg per mL *p*-anisidine yielded a 7.0 mg per mL PPA solution, with the concentration of PPA being determined by measuring the thickness of a film drop cast from a known volume and deposited on a known surface area, assuming a polymer density of 1.0 g cm^−3^.

#### Film preparation

Dialyzed PSI and dialyzed PPA solutions were mixed at weight percentages ranging from 0 to 16.7% PSI and sonicated for 10 min. Gold substrates (or glass) were cleaned with DI water and ethanol and then dried with a nitrogen stream. Electrochemical masks with a 0.27 cm^2^ punched cavity were mounted atop clean gold substrates. PSI–PPA mixtures were then drop cast as shown in Scheme 1 at varying loadings, yielding multiple film thicknesses, compositions, and surface coverages (mg cm^−2^).

### Characterization techniques

Infrared spectra were obtained using attenuated total reflectance Fourier transform infrared (ATR-FTIR) spectroscopy with a Thermo Nicolet 6700 FT-IR spectrometer equipped with a liquid-nitrogen-cooled mercury–cadmium–telluride (MCT) detector and Smart iTR ATR attachment with a diamond crystal plate. The spectra were collected in the region of 1800–700 cm^−1^ over 256 scans at 2 cm^−1^ resolution and processed using the OMNIC software. The diamond crystal in the FT-IR was exposed to open air to obtain the background for all data collection.

Nuclear magnetic resonance (NMR) spectra were recorded on a Bruker 600 MHz spectrometer equipped with a cryoprobe. The samples were dissolved in DMSO-*d*_6_ in a 3.0 mm tube to a final concentration of ∼30 mg mL^−1^. The spectra were acquired at 25 °C (298 K). NMR chemical shifts were reported as *δ* values in ppm relative to the residual solvent peak (DMSO, *δ*_H_ 2.50 ppm/*δ*_C_ 39.5 ppm). Proton and carbon assignments were inferred from 2D NMR techniques, such as heteronuclear single quantum coherence (HSQC) and heteronuclear multiple bond correlation (HMBC), as shown in the ESI.†

Scanning electron microscopy (SEM) of pristine PPA and 16.7% PSI/PPA films were taken using a Hitachi S-4200 SEM at an accelerating voltage of 2 kV and a 100 pA maximum current. Both films were drop cast onto gold to a total surface coverage of 3.0 mg cm^−2^.

Profilometry was used to determine the thickness of drop cast films as well as the concentration of the PSI and PPA solutions. Profilometric thickness was determined using a Bruker Dektak 150 stylus profilometer with measurements performed over 7000 μm across the entire film using a stylus with a 12.5 μm radius, applying 2.0 mg force, and employing a hills-and-valleys detection mode.

An Ossila four-point probe system (product T2001A3-UK) was used to measure the conductivity of PPA films on a glass substrate. A ∼30 μm film of dialyzed PPA was drop cast onto a glass substrate. The measurements were recorded at 128 samples per point, a target current of 1 nA, and a maximum voltage of 5 V. The applied current resulted in a total of 15 repeat values of sheet resistance, resistivity, and conductivity for each sample measured with the averages and standard deviations of three data sets as the reported results.

Thermogravimetric analysis (TGA) was used to assess PPA's thermal stability and heating response. ∼2.0 mg of PPA were added to a ceramic sample pan after multiple drop casting cycles of the dialyzed PPA solution. The pan was then placed in an STA-i 1000 thermogravimetric analyzer, and air was introduced into the furnace. PPA was heated to 85 °C at a rate of 5 °C min^−1^, then from 85 to 105 °C at a rate of 2 °C min^−1^ and held for 30 min to remove entrapped water within the polymer; then, the sample was heated at a 10 °C min^−1^ ramp to 600 °C.

#### Photoelectrochemical characterization

Photochronoamperometry (PCA) was performed using a CH660a potentiostat (CH Instruments, Austin, TX) with a three-electrode assembly and a Faraday cage. The working electrode consisted of film-coated gold substrates, the reference electrode was Ag/AgCl, and the counter electrode was a platinum mesh. A cold light source (Leica KL 2500 LCD) emitting a light intensity of 100 mW cm^−2^ at the surface with a spectral range of 380–790 nm, was used during the 20 s light illuminations on the film. The photoelectrochemically active area during experiments was constrained to the 0.27 cm^2^ drop cast region. For all films, the open circuit potential (OCP) was first measured in the dark, and the resulting value was then applied to the film during the collection of *i*–*t* curves. Biasing the working electrode with the dark OCP allows the background current to start at ∼0 A, eliminating the contribution of the inherent potential differential in the system, and attributing any further change in current during the 20 s illumination period to reactions within the film and at the electrode surface. PCA measurements were performed using a redox mediator at two distinct concentrations. The more dilute solution contained 0.1 mM potassium hexacyanoferrate(ii) trihydrate K_4_[Fe(CN)_6_]·3H_2_O, 0.1 mM potassium hexacyanoferrate(iii) K_3_[Fe(CN)_6_], and 100 mM KCl, whereas the more concentrated mediator solution had the same KCl concentration but contained 1.0 mM of K_4_[Fe(CN)_6_]·3H_2_O and 1.0 mM of K_3_[Fe(CN)_6_].

UV-Vis spectroscopy was performed with a Varian Cary 5000 UV-VIS-NIR Spectrophotometer following two distinct experimental procedures. Transmittance–absorbance spectral scans were performed from 800 to 200 nm at a rate of 600 nm min^−1^ and a sampling frequency of 1 nm. For Fig. 3b, a quartz cuvette was used, and the spectrum of PPA in solution was obtained by diluting chemically polymerized and dialyzed PPA by 20-fold in DI water, whereas *p*-anisidine was measured at a concentration of 0.1 mM, with DI water used as background for both measurements. In Fig. 6, the percent transmittance (%*T*) through PPA films drop cast on glass was measured. Samples were mounted to a transmittance–absorbance holder and a 100% transmittance blank sample (uncoated glass) was used as a baseline for all PPA samples. The %*T* for the two distinct *chlorophyll a* characteristic absorbance wavelengths (440 nm and 675 nm) were used to measure the light passing through the film for PSI-relevant wavelengths.

## Results and discussion

### Polymer characterization

#### PPA film composition and conductivity

PPA was prepared by oxidative polymerization of *p*-anisidine with ammonium persulfate, similar to the method of Rodrigues de Oliveira *et al.*^34^ The monomer had a conversion of ∼80% and the polymer concentration in the resulting aqueous solution was ∼7.0 mg mL^−1^. The conductivity of a ∼30 μm thick film of PPA on glass was measured with a four-point probe as (3.1 ± 0.1) × 10^−5^ S m^−1^, which is ∼1000 times higher than the conductivity reported for PPA dry pellets with an impedance analyzer by Rodrigues de Oliveira *et al.*^34^ The higher conductivity here is attributed to the different method of measurement and the unique compositional ratios of monomer and oxidant in our study. Rodrigues de Oliveira *et al.* observed that the ratio of monomer to oxidant had a significant effect on polymer structure and pellet conductivity.^34^

**Fig. 1:**
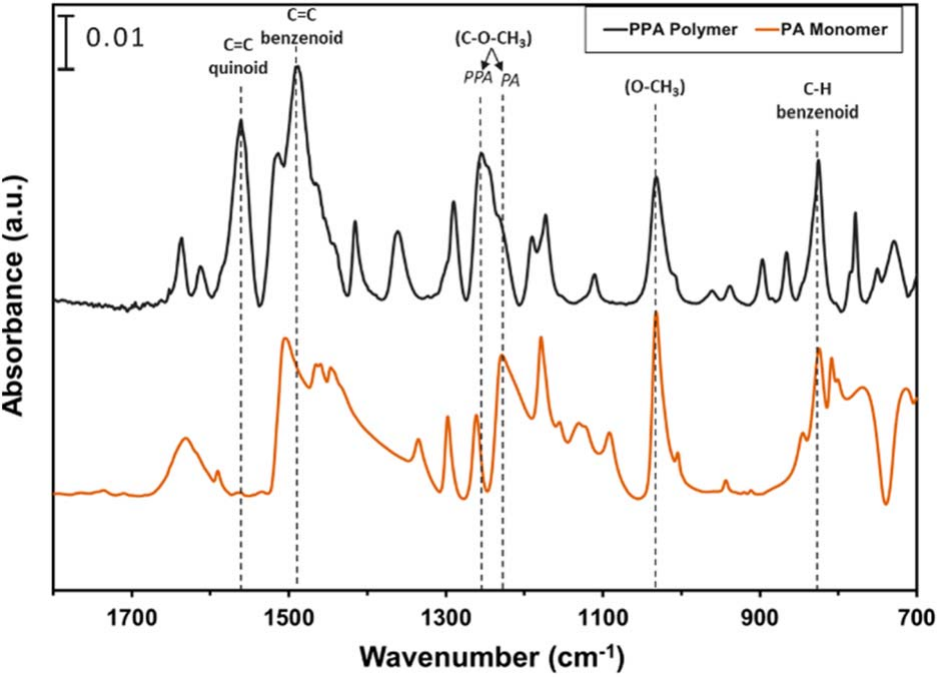
ATR-FTIR spectra of *p*-anisidine and PPA with characteristic peaks noted. The spectra are offset vertically for clarity.

ATR-FTIR spectroscopy was used to compare the IR peaks of the *p*-anisidine (PA) monomer *vs.* the PPA polymer, and to further validate a successful polymerization. Fig. 1 shows how multiple peak intensities differ between the IR spectrum of the monomer (bottom) and the polymer (top) and assigns some of the main characteristic peaks to their appropriate molecular imprint. The peaks at 825 cm^−1^ and 1030 cm^−1^ are attributed to the out of-plane *γ*(C–H)^40^ and *ν*(O–CH_3_),^41^ respectively, and are common in both the monomer and the polymer. The absorbance peak at 1250 cm^−1^ for PPA is attributed to *ν*(C–O–CH_3_), which exhibits a slight shift from that same bond frequency that is reported in literature and identified for *p*-anisidine at 1235 cm^−1^.^34,42^ PPA-specific bonds are identified at 1490 cm^−1^ and 1560 cm^−1^ for C=C benzenoid and C=C quinoid, respectively, with these bonds only appearing when repeat units are present.^34,35^ The ratio of the quinoid and benzenoid in the polymer reveals a comparable ubiquity of these bonds in the polymer backbone.

**Fig. 2:**
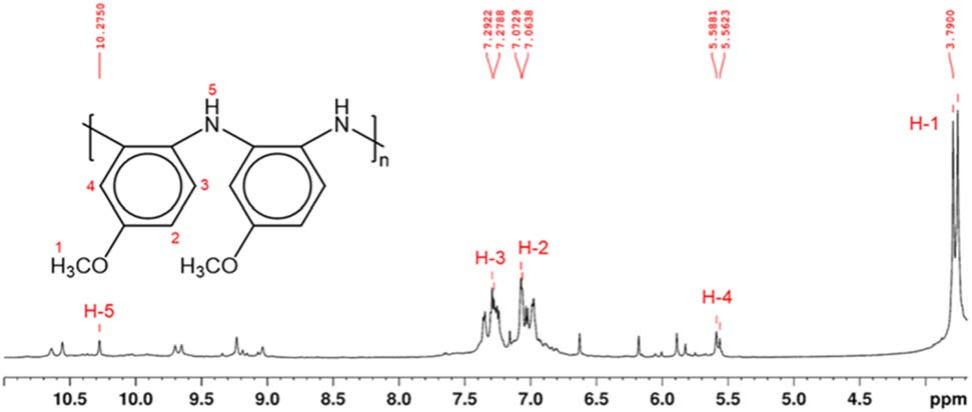
^1^H NMR spectrum and proposed molecular structure of PPA, as determined by 2D NMR analysis in Fig. S2 and S3.† The molecular structure shows only the benzenoid structures, while the quinoid structure has been omitted for clarity. Hydrogen atoms are enumerated in the molecular structure and their corresponding peaks in the ^1^H NMR spectrum are labeled in red.

Structural characterization of PPA was conducted using NMR spectroscopy. In polymeric materials, similar functional moieties in different stereochemical and compositional environments experience distinctive chemical environments, thereby inducing variations in the chemical shifts of various ^1^H nuclei.^34^ As a result, the spectrum of a polymer can exhibit overlapping neighboring signals with a similar spectral profile, as observed in the ^1^H NMR spectrum of PPA in Fig. 2. In this spectrum, the main peaks at *δ* 7.0–7.4 ppm and *δ* 3.8 ppm correspond to the aromatic (H-2, H-3) and the methoxy (H-1) protons, respectively.

Since the polymerization reaction of *p*-anisidine can only occur between the amine group and the *ortho* or the *meta* position of the amine in the adjacent monomer, the detailed structural elucidation of the PPA requires further investigation. To discern the precise polymerization route from PA to PPA, we have used two-dimensional NMR HSQC and HMBC to establish the resonance signals due to the neighboring protons and assign proton signals that could not be identified solely by ^1^H NMR. Fig. S2 and S3† summarize the most important HSQC and HMBC correlations. These results suggest a predominant polymerization route with the amine group of a monomer bonding to the *ortho*-position of the amine of an adjacent repeat structure, as shown in the inset of Fig. 2. The greater activation of the carbon at the *ortho*-position of the amine group as compared to that by the methoxy group results in this dominant site for the polymerization.^43^ Furthermore, the overlapping of the chemical shift signals between *δ* 6.9 and 7.4 observed in the ^1^H NMR in Fig. 2 suggests a non-crystalline arrangement of the polymer, consistent with the proposed chemical structure. In agreement with the IR spectrum of PPA, signals due to the presence of quinoid structures are also identified in the 2D NMR.^34^ Scheme S1† combines results from the IR and NMR data into a PPA molecular structure with both benzenoid and quinoid structures present.

#### TGA and UV-Vis

**Fig. 3:**
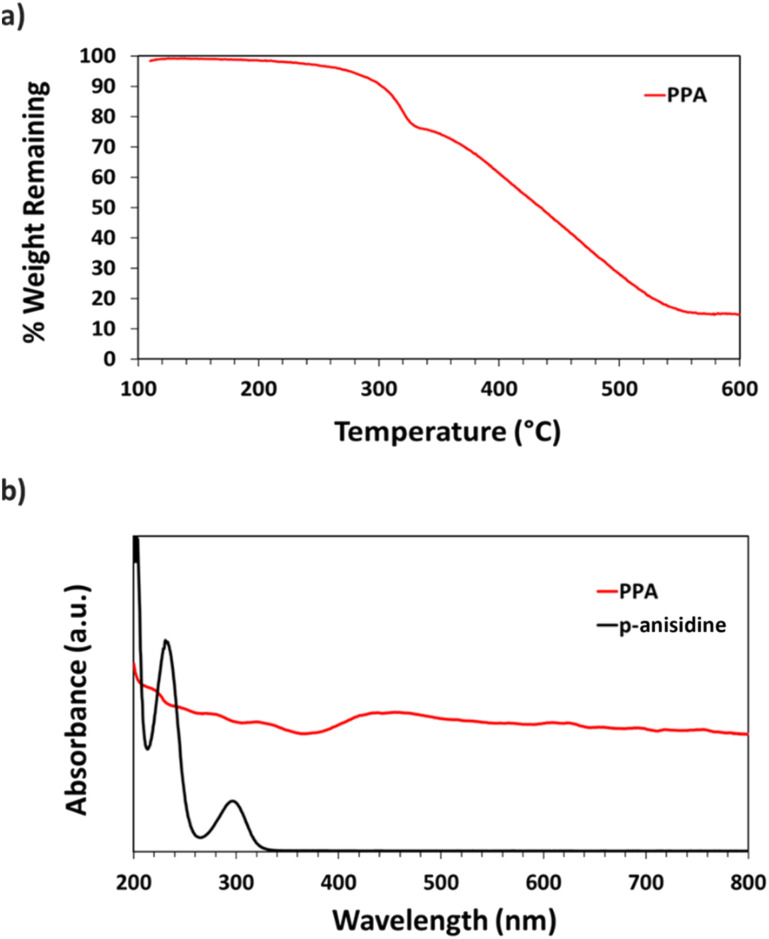
(a) TGA thermograph of PPA (b) UV-Vis spectra of *p*-anisidine and PPA.

Thermogravimetric analysis (TGA) was used to quantify the thermal decomposition of PPA with increasing temperature. Fig. 3a shows the TGA curve of PPA, starting at 105 °C so that residual water is removed and that the weight loss could be attributed to the polymer itself. The thermal behavior of PPA resembles that of polyaniline and POA in that we observe three main stages of decomposition within the 105–600 °C temperature range.^44,45^ The first and second stages of decomposition between 105 and 320 °C are attributed to the loss of the HCl dopant from the polymer chain, while the third stage reflects the full degradation and decomposition of the polymer backbone.

UV-Vis spectroscopy was used to obtain the absorbance spectra of *p*-anisidine and PPA in aqueous solution. Fig. 3b shows the differences between the absorbance spectrum of the monomer *versus* that of the polymer. *p*-Anisidine, when diluted in DI water at 0.1 mM yields three main absorbance peaks in the ultraviolet range, but none in the visible range. The three peaks present at 300 nm, 233 nm, and 202 nm are in accordance with a previously reported *p*-anisidine UV-Vis spectrum.^41^ The UV-Vis spectrum of chemically polymerized and dialyzed PPA was obtained by diluting the stock PPA solution by 20-fold in DI water, which resulted in a PPA spectrum that is significantly distinct from its monomer, with light absorbance all throughout the visible range, consistent with its dark hue. Peaks for PPA in the UV region occur at 334 nm, 278 nm, and 222 nm.

### PSI–PPA biohybrid films

Prior to drop casting, PSI and PPA were mixed at various concentrations, and the resulting solutions were used to prepare PSI–PPA composite films by drop casting in a well-ventilated hood, as shown in Scheme 1. PSI was introduced at dilute concentrations varying between 0 and 16.7 wt% in mixed solutions.

#### IR spectroscopy PSI–PPA

**Fig. 4:**
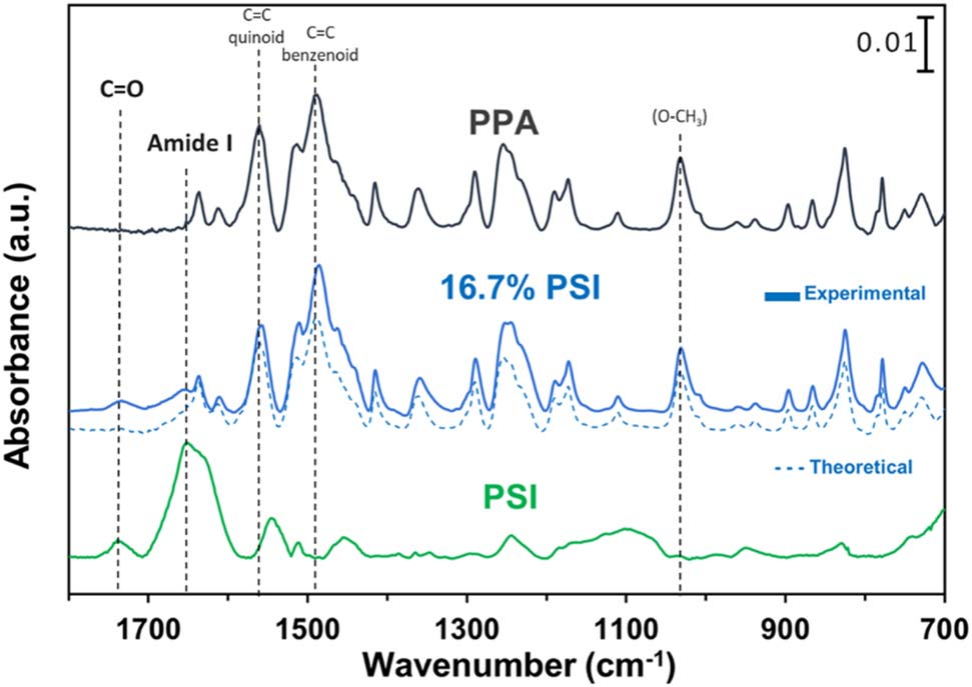
ATR-FTIR spectroscopy of PSI (bottom), 16.7 wt% PSI in PPA with a theoretical IR plot shown in dashed lines (middle), and chemically polymerized PPA (top) where characteristic peaks are noted for PSI and additional dotted lines tracking key PPA peaks. The spectra were offset vertically for clarity.

The contribution of each component in the composite films was investigated using IR spectroscopy. Fig. 4 shows the spectra of PSI, PPA, and a composite PSI–PPA film at 16.7% PSI. Additionally, the dashed line in Fig. 4 represents the theoretical arithmetic mean of 16.7% of the IR intensity of PSI and 83.3% of the PPA IR intensity, resulting in a curve that closely resembles that of the experimentally reported values, further validating the presence of PSI at this dilute concentration within the composite film. Three of the dotted lines correspond to previously identified PPA peaks from Fig. 1, but two more characteristic peaks are identified for PSI alone and are tracked to peaks present in the composite. While the IR spectrum displays an apparent domination of PPA peaks in the PSI–PPA composite at this ratio of concentrations, it also shows contribution of the PSI peaks at two distinct wavenumbers. The amide I peak at ∼1655 cm^−1^ is due to the protein's C=O vibrational modes of the amides, and the peak at 1750 cm^−1^ is due to carboxylic acids within PSI as well as the ester C=O of PSI's *A*_0_ cofactor.^46,47^

#### Film morphology

**Fig. 5:**
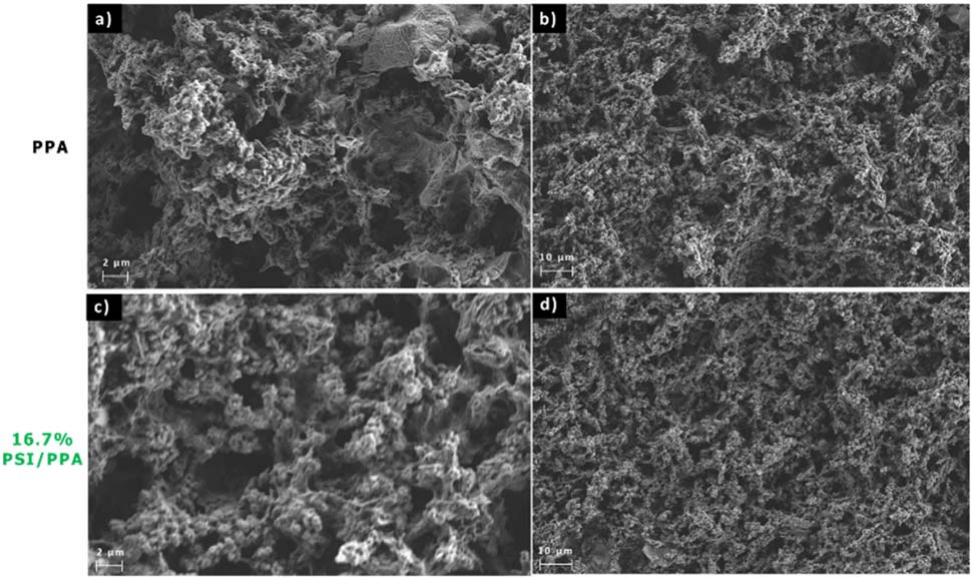
SEM images of (a) and (b) pristine PPA and (c) and (d) 16.7% PSI.

We used SEM to characterize the morphology of pure PPA films (Fig. 5a and b) and a 16.7% PSI composite film (Fig. 5c and d). Fig. 5a at a 2 μm scale shows agglomerated globular structures, irregular pores, and a non-uniform surface, all in accordance with a previously reported morphology for polyaniline and its derivatives.^35,48^ The presence of both elongated needles and globular morphologies in the polymer films has been reported by Rodrigues de Oliveira *et al.* to be consistent with the C–N bonds forming during polymerization at the carbon in the *ortho* position of the repeating structure. Thus, the polymer morphology from Fig. 5a further validates the addition of the repeat structure at the *ortho* position of the amine in the aromatic ring and aligns with the NMR data from Fig. 2.^34^Fig. 5b was taken at lower magnification with a 10 μm scale, showing a continuous film with porous morphology across a larger area. Fig. 5c and d are of the composite film at 2 μm scale and 10 μm scale, respectively, showing a morphology that is similar to that of pristine PPA. The apparent similitude is not surprising given that the size of PSI extracted from spinach has 16 × 12 nm^2^ dimensions,^49^ making the identification of single proteins impossible at this scale. However, a closer examination reveals slight differences in the images at the 2 μm scale. When comparing Fig. 5a and c, we notice that the image is significantly less crisp for the 16.7% PSI film (Fig. 5c) than for the pristine PPA film. Obtaining SEM images of PSI-containing films is typically challenging due to the insulating nature of the protein, often requiring gold sputtering for improved resolution.^5^ Other than charging effects, Fig. 5a and c suggest that the presence of PSI does not notably affect the film morphology, which is expected if there are no phase-separated regions of aggregated protein. Thus, PSI and PPA appear to be mixed within the composite film without microscale protein aggregation. On the larger 10 μm scale, differences between Fig. 5b and d are even more challenging to pinpoint with the two films exhibiting a multitude of similarities in their bulk morphological properties.

#### Profilometry and UV-Vis

**Fig. 6:**
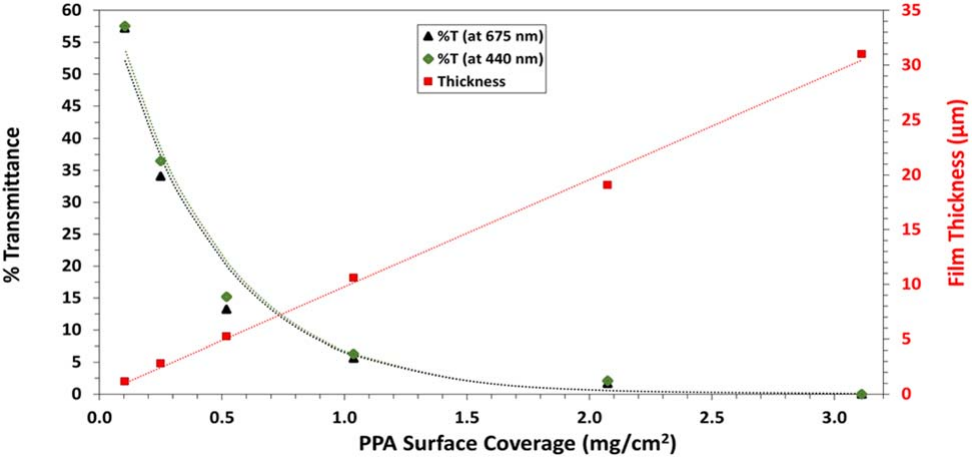
Percent light transmittance at *chlorophyll a* absorption wavelengths (440 nm and 675 nm) (left axis) and profilometric film thicknesses (right axis), all with varying PPA surface coverage. The red dotted line represents the linear fit through the film thickness data points. The green and black dotted lines represent the best fit model based on Beer–Lambert law for %*T* at 440 nm and 675 nm, respectively.

The overarching goal of this work is to fabricate photoactive PSI–PPA composite films, and in those efforts, we first quantify the %transmittance (%*T*) of light through PPA films at wavelengths corresponding to *chlorophyll a* (*chl a*) absorbance in the visible light. For effective photoexcitation of PSI proteins at the P_700_ active site, the incident white light must penetrate the film to PSI proteins far from the outer surface. Fig. 6 shows the %*T* through PPA films drop cast on glass substrates at *chl a*-relevant wavelengths (440 nm and 675 nm), as well as the profilometric thickness of the film, both at varying PPA surface concentrations ranging between 0.1 and 3.1 mg cm^−2^. The thickness follows a near linear behavior and increases from ∼2 μm at 0.1 mg cm^−2^ PPA to ∼31 μm at 3.1 mg cm^−2^. Transmittance is inversely correlated to PPA surface coverage and decreases from ∼58 %*T* at the lowest PPA surface coverage to ∼0.01 %*T* at the highest coverage. In between these two extremes, %*T* follows Beer–Lambert's law that translates to %*T* scaling with *a* × 10^−[PPA]^, where “*a*” is a constant determined from the fit. Since the %*T* approaches zero at 3.1 mg cm^−2^, the surface concentration was kept below that value for all composite PSI–PPA films.

#### Photochronoamperometry

**Fig. 7:**
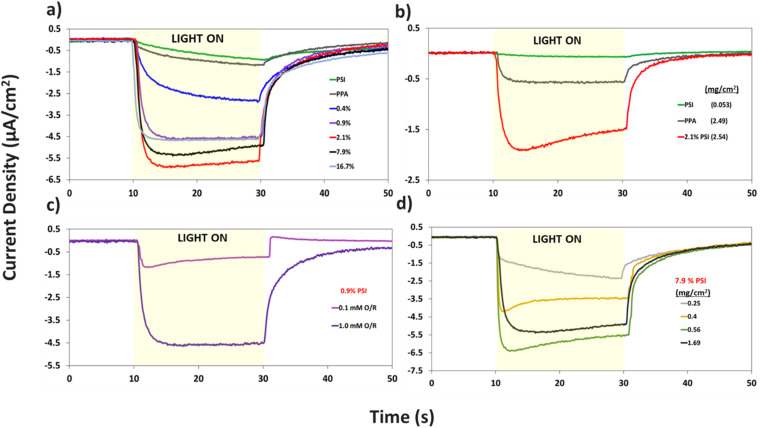
Photochronoamperometric measurements of (a) pristine PPA, pristine PSI, and composite PSI–PPA films at varying wt% PSI, all measured in 1 mM redox mediator concentration, (b) PSI, PPA, and 2.1% PSI showing the synergistic photoactivity between the two mixed components at 0.1 mM redox mediator concentration. (c) 0.9% PSI measured in varying redox mediator concentrations, (d) 7.9% PSI in 1 mM redox mediator concentration at varying film loadings showcasing the effect of thickness on the film's photoactivity. All photocurrents fall within ±35% of data collected for a minimum of four repeat measurements on two different electrodes.

PCA measurements were performed to investigate the photoactivity of PPA, PSI, and composite PSI–PPA films on gold, as well as the effect of redox mediator concentration and film thickness on the measured current in the presence of white light. All PCA experiments were performed in a three-electrode setup with a film-coated gold electrode, using ferricyanide (Fe(CN)_6_^3−^)/ferrocyanide (Fe(CN)_6_^4−^) as the redox couple to mediate the electron transfer. Changes in current *vs.* time caused by 20 s long light illuminations at OCP reflect the ability of the film to absorb light that is used to excite electrons, transfer those electrons within its matrix, and exchange them with the gold substrate and the redox mediator. Fig. 7a represents the PCA measurements of PSI, PPA, and PSI–PPA composite films in 1 mM redox mediator. Importantly, PPA is shown for the first time to be photoactive and can produce ∼1.2 μA cm^−2^ of photocurrent density under these conditions of white light and mediator. The pristine PSI film, which is at the same protein loading as a 7.9% composite film, exhibits a slow rise in current similar to PPA, but produces the lowest photocurrent of ∼0.9 μA cm^−2^, relating to its insulating nature and its inability to effectively transfer excited electrons to other proteins within the film.^50^ As such, the PSI film relies solely on the redox mediator for electron exchange.^50^ As we introduce PPA and its electrical conductivity and electron transport capability, the photocurrent increases sharply with increasing PSI loading to reach a maximum value of ∼6 μA cm^−2^ for the composite film at 2.1% PSI. The photocurrent then decreases gradually with increasing PSI loadings within the composite film to a current density of ∼4.5 μA cm^−2^ at 16.7% PSI. This maximum photocurrent at low PSI loading is consistent with previous results of PSI and other conducting polymers,^5,51^ showing that a tradeoff exists between PSI loading for enhanced light absorption and inter-film conductivity. Dilute PSI films with high protein–polymer interfacial areas can rapidly transfer excited electrons to neighboring conductive molecules. The porous structure of these composite films apparent in Fig. 5d allows for further film-redox mediator interfacial area, supporting electron transfer within the three-electrode electrochemical setup herein. Although PPA exhibits lower electrical conductivity than many of the other commercially available conducting polymers, its energy alignment with P_700_ generates photocurrents that are comparable to other PSI-conducting polymer films on metal electrodes.^5,51,52^

Additional analyses on the data in Fig. 7a are shown in Fig. S4 and S5† to inspect the role of PSI on the photocurrent of the composite film, and to further examine the effect of PSI loading on the time elapsed for the composite films to reach maximum photocurrent (*i*_max_). Fig. S4† shows that prolonged exposure of PSI to UV light reduces its measured photocurrent by ∼4×. When used in a composite film, this partially deactivated protein (PSI_d_) in turn decreases the photocurrent of the 2.1% composite film by ∼3×, validating the integral role PSI plays in the photoactivity of the films. Fig. S5a and b† show the effect of PSI loading on the rate of photocurrent rise, namely with PSI loadings of 0.9% or greater supporting rapid electron transfer within the film and yielding much faster times for the photocurrent to reach its maximum value (*i*_max_).

The same protein loading in the pristine PSI film was mixed with PPA to fabricate the 7.9% composite film and produces a photocurrent that is ∼3 times higher than that of each component (Fig. 7a). Thus, there exists a synergistic effect in photocurrent upon mixing the two components. This phenomenon is further validated in Fig. 7b, where even at a lower 0.1 mM redox mediator concentration, we observe the 2.1% composite film yielding a much greater response to light than each component alone. The effect of redox mediator concentration is emphasized in Fig. 7c where at 0.9% PSI, the same composite film yields a photocurrent that is ∼5× larger when interfaced with the more concentrated redox solution. Comparison of Fig. 7a with S6a† further illustrates that distinction by showing that photocurrents at all PSI loadings are increased at higher mediator concentration (Fig. S6b†). Since both PPA and PSI can accept electrons from reduced mediator species, the concentration of the redox mediator becomes paramount in supplying electrons to the photoactive layers, and subsequently, the working electrode.

Holding the concentration of the redox mediator at 1.0 mM and the PSI loading at 7.9%, we show the effect of composite film surface coverage to illustrate the impact of film thickness on the resulting photocurrents (Fig. 7d). Photocurrents increase with increasing PSI–PPA loading to reach a maximum of ∼6.5 μA cm^−2^ at 0.56 mg cm^−2^ total surface coverage (0.44 mg cm^−2^ PPA and 0.12 mg cm^−2^ PSI) and then trickle back down to ∼5.5 μA cm^−2^ at 1.69 mg cm^−2^ total surface coverage (the same loading used for 7.9% PSI in Fig. 7a). The maximum photocurrent at intermediate loadings is attributed to the ability to achieve a continuous, defect-free film of the composite material while still allowing sufficient light transmission. In summary, these PCA results support the photoactivity of all measured PSI–PPA composite films and of PPA alone, as well as a synergistic effect between the polymer and the protein where composite films produce much higher photocurrents than each component alone does.

**Fig. 8:**
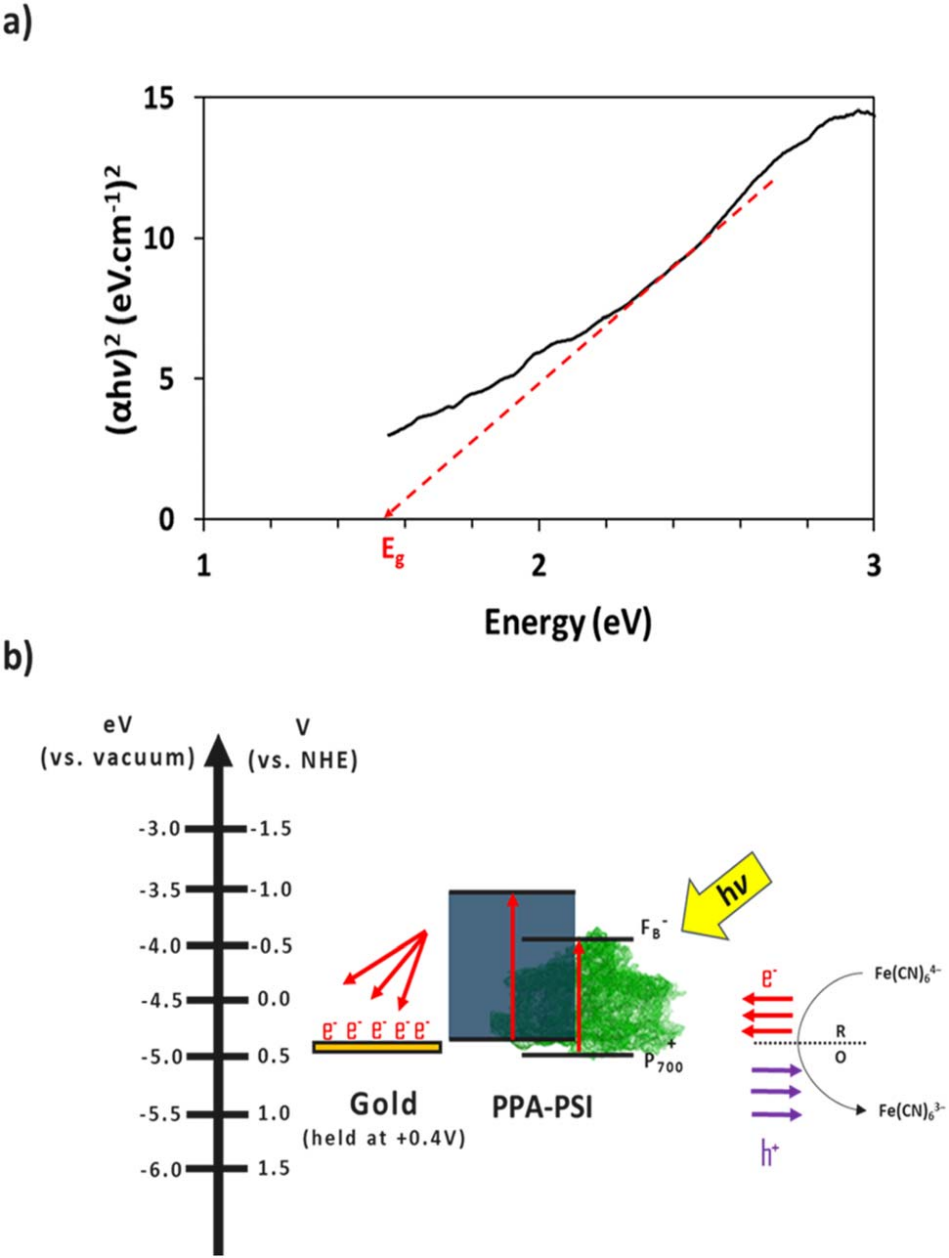
(a) Tauc plot of PPA. The intersection of the dashed line with the *x*-axis indicates PPA's band gap. (b) Schematic of the proposed electron transfer mechanism between the redox mediator, the composite film, and the gold substrate, neglecting recombination. Electron movement is represented in red and hole movement is represented in purple.

The electron transfer within the three-electrode setup described herein is further investigated to better understand the underlying electron transfer mechanism for drop cast PSI–PPA films. PPA is a conducting polymer behaving as an organic semiconductor, so the parameters that are important to identify in order to understand the polymer's role in the electron transfer within the composite film are the electronic band gap (*E*_g_), the energy of its highest occupied molecular orbital (HOMO), and of its lowest unoccupied molecular orbital (LUMO).^53^ The formal/charge band gap could differ from the optical band gap of PPA, but the electronic and optical properties, including HOMO–LUMO energy gaps of this organic semiconducting polymer can be accurately estimated by UV-Vis.^54^ The absorption edge of an organic semiconductor can be deduced from the UV-Vis absorption profile of the polymer and follows the relationship in eqn (1):^55^1
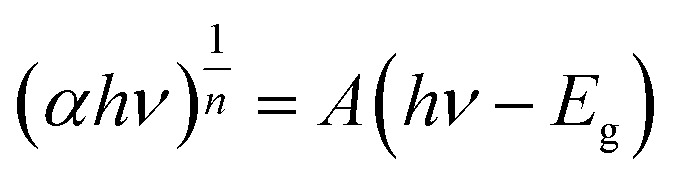
where *α* is the energy-dependent absorption coefficient, *h* is the Planck constant, *ν* is the photon frequency, *A* is a constant, and *E*_g_ is the band gap energy. The *n* factor is either equal to ½ or 2 depending on whether the electron transition in the band gap is direct or indirect, respectively. PPA follows a direct band gap,^40^ and the corresponding Tauc plot based on the absorbance and *n* = 1/2 is shown in Fig. 8a. The band gap is identified to be *E*_g_ = 1.55 eV, slightly larger than the one reported for polyaniline (1.38 eV).^56^ The HOMO of PPA is estimated at +407 mV *vs.* NHE from the first oxidation peak in the CV of PA shown in Fig. S1.† The electrochemical oxidation of the monomer is not expected to deviate largely from that of the polymer, as electron hopping occurs from one repeat unit to the other in the polymer backbone. Thus, for PPA that is chemically and oxidatively polymerized with ammonium persulfate as the active oxidant, and Cl^−^ as the counterion, we estimate an *E*_g_ ∼ 1.6 eV, a HOMO = +0.407 V, and a LUMO = −1.05 V at ambient temperature and pressure, noting that the band gap energy of organic semiconductors can change with the electronic band structure depending on the pressure, temperature, and polymer synthesis conditions.^54,57,58^

The combination of the electrical properties of PSI, PPA, the redox mediator, and gold yields the light-driven electron transfer mechanism proposed in Fig. 8b. In this wet electrochemical system, the electrons are mainly supplied by the reduced redox mediator ferrocyanide (Fe(CN)_6_^4−^), whose concentration in the bulk plays an integral role in the resulting photocurrent of the composite PSI–PPA films. The greater the concentration gradient, and the more reduced ferrocyanide species in solution, the more favorable the conditions for electrons to move toward the working electrode (WE). The gold substrate is held at the OCP of ∼+0.2 V *vs.* Ag/AgCl (∼+0.4 V *vs.* NHE) measured in the dark, reducing the over-potential between the redox mediator and the gold, and limiting but not eliminating gold/mediator electron transfer.

The P_700_ active site and PPA's HOMO are both close in energetics to the electron donating potential of ferrocyanide but with P_700_ having a ∼100 mV of underpotential to PPA and more favorable energetics to be reduced by ferrocyanide. PPA can still receive an electron from ferrocyanide and generate a photocurrent as shown in Fig. 7a, but the relative level of effectiveness is well below that of the composite films. Combining PSI and PPA yields a synergistic effect on photocurrent for two main reasons. First, the PSI–PPA composite films overcome the insulating nature of PSI by introducing a conducting polymer. Second, the composite films retain PSI's ability to produce excited electrons that reside at much higher energy levels than those of the reduced mediator. The excited PSI electrons can subsequently reduce PPA more effectively at its HOMO than in the case of PPA/mediator, enabling the PSI–PPA composite to far exceed the photo-driven performance of each component alone.

## Conclusions

Chemically and oxidatively polymerizing *p*-anisidine in acidic conditions forms C–N bonds at the carbon in the *ortho* position of the repeating structure yielding a PPA polymer that is thermally stable, photoactive, and conductive with semiconducting properties. Combining PPA with PSI does not denature the protein or deactivate its photo-response. Drop cast composite films of PPA and PSI exhibit a porous morphology that is similar to that of the polymer alone. The porous morphology provides high interfacial areas for charge transfer with diffusible redox species.

The PSI–PPA films were found to be photoactive, with photocurrents depending on the loading of PSI, the overall loading/thickness of the composite, and the concentration of redox mediator. The optimum photocurrent at a redox mediator concentration of 1.0 mM was for a composite film with a dilute PSI concentration of 2.1 wt%. Previously, we observed optimum photocurrent at ∼10 wt% PSI when spin coated with PEDOT:PSS.^5^ These combined results show that a tradeoff exists between PSI loading for enhanced light absorption and inter-film conductivity, with the insulating protein introducing a hindrance to the conduction of electrons within the film at high loadings but still allowing improved light absorption at low concentrations. This conclusion may have broader implications for various biohybrid protein–polymer systems. In instances involving other bulky photoactive proteins, such as photosystem II, which similarly possesses electrically insulating properties, optimal performance in photocurrent optimization of biohybrid films may be achieved at dilute protein concentrations.

The maximum photocurrent for a 7.9% PSI composite film was at a surface coverage of 0.56 mg cm^−2^, an intermediate loading. To obtain maximum photocurrents at a given %PSI, PSI–PPA films need to coat the gold substrate enough to achieve a continuous and defect-free film, while still allowing sufficient light to transmit through the composite layers deeper within the film. For all PSI loadings reported, the higher redox mediator concentration (1.0 mM) resulted in higher photocurrents. A higher concentration of mediator species is needed to maintain sufficient electron flux into the composite film throughout the porous network. In this work, we have shown that, regardless of PSI loading or mediator concentration, the photocurrent of the composite films is much greater than that of each pure component. This synergistic effect is consistent with a high interfacial area between PSI and PPA to facilitate the transfer of photoexcited electrons from the protein to neighboring conductive molecules.

## Author contributions

M. A. Nabhan: investigation, methodology, and writing – original draft. A. V. Cordova-Huaman: investigation, and writing – original draft. D. E. Cliffel: conceptualization, writing – review and editing. G. K. Jennings: conceptualization, supervision, and writing – review and editing.

## Conflicts of interest

There are no conflicts to declare.

## Supplementary Material

NA-006-D3NA00977G-s001
