# Supplementary material for: Interfacing poly(p-anisidine) with photosystem I for the fabrication of photoactive composite films
Source: Nanoscale Adv. 2023 Dec 22;6(2):620–9. doi: 10.1039/d3na00977g (PMC10790974; doi:10.1039/d3na00977g)
Supplement: NA-006-D3NA00977G-s001 [file NA-006-D3NA00977G-s001.pdf]

Electronic Supplementary Material (ESI) for Nanoscale Advances.
This journal is © The Royal Society of Chemistry 2023

Electronic Supplementary Information

Interfacing Poly(p-anisidine) with Photosystem I for the Fabrication of Photoactive Composite Films

Marc A. Nabhan[1], Allison V. Cordova-Huaman[1], David E. Cliffel[2], G. Kane Jennings[1,*]

[1] Department of Chemical and Biomolecular Engineering, Vanderbilt University, Nashville, Tennessee 37235-1604, United States

[2] Department of Chemistry, Vanderbilt University, Nashville, Tennessee 37235-1822, United States

*Corresponding author.

E-mail: kane.g.jennings@vanderbilt.edu

Cyclic Voltammetry

Cyclic voltammetry (CV) was performed to electropolymerize p-anisidine and obtain the energetics at which redox reactions occur within the resulting PPA polymer film. A three-electrode system and a CH660a potentiostat (CH Instruments, Austin, TX) equipped with a Faraday cage were used. The working electrode was comprised of a clean gold substrate and was used in conjunction with a Ag/AgCl reference electrode and a platinum mesh counter electrode.

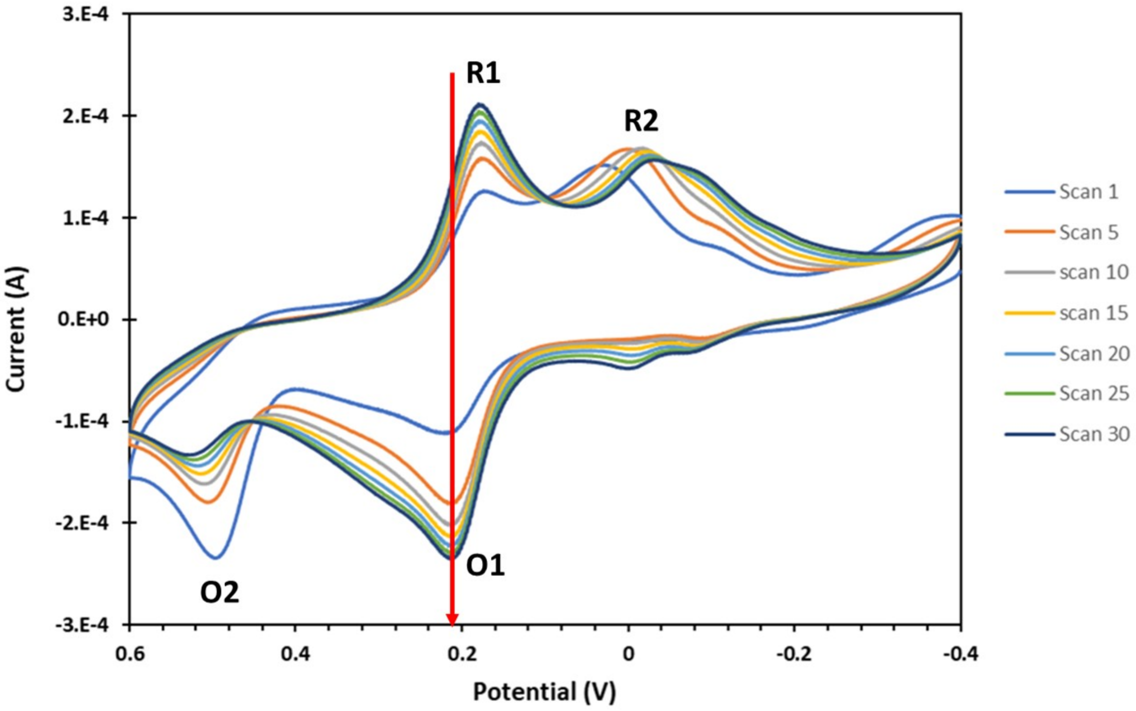

Figure S1. Cyclic voltammetry of p-anisidine in solution. The red arrow indicates the first oxidation peak for p-anisidine in the +V direction. R1 and R2 represent the two reduction peaks. O1 and O2 are labels for the two oxidation peaks.

The CV shown in Figure S1 was collected in a solution of 0.25 mM p-anisidine, 3.0 M KCl, and 1.0 M sodium acetate/acetic acid buffer for 30 scans. The CV indicates a dominating first oxidation peak (O1) at +0.210 V vs Ag/AgCl (+407 mV vs NHE), which was assigned as the energy level for

the highest occupied molecular orbital level (HOMO) of the polymer. Running a similar experiment as the one yielding the CV in Figure S1, but in the voltage range of -0.4 V to +0.35 V vs Ag/AgCl to avoid generating peak O2, shows that peak O1 does not align with the first reduction peak R1 and that the redox reaction is irreversible. The electropolymerization of p-anisidine is a 2-electron process with O1/R2 and O2/R1 being the two redox couples involved in this process.

NMR Spectroscopy

2D NMR experiments were employed to determine the chemical structure of PPA. 2D ^{1}H–^{13}C HSQC and HMBC NMR experiments were recorded on a Bruker 600 MHz spectrometer (600 MHz and 150 MHz for ^{1}H and ^{13}C NMR, respectively). HSQC experiments were acquired using a 1024 x 256 data matrix and 32 scans while the HMBC spectrum was acquired using a 2048 x 128 data

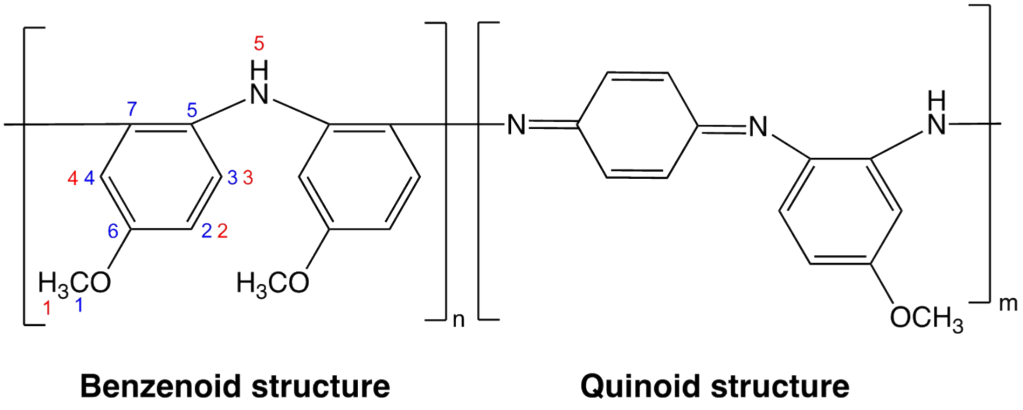

Scheme S1. PPA Molecular drawing including both the benzenoid and quinoid structure. The benzenoid structure is enumerated with corresponding numerical assignments used in NMR with hydrogen and carbon atoms enumerated in red and blue, respectively.

matrix and 128 scans. The data collected from both 2D NMR experiments was processed using a

π/2 shifted squared sine window function, with the HSQC data displayed with CH/CH_3 signals phased positive and the HMBC displayed in magnitude mode.

Proton resonance signals that could not be assigned by the ^{1}H NMR spectrum alone were

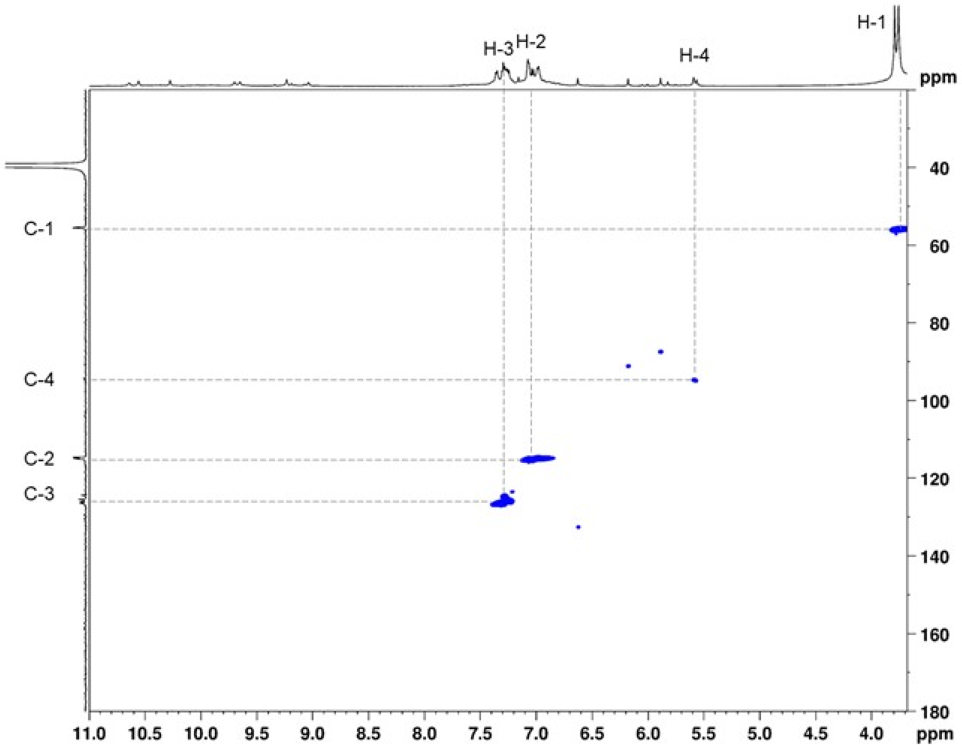

Figure S2. 2D HSQC of PPA in DMSO.

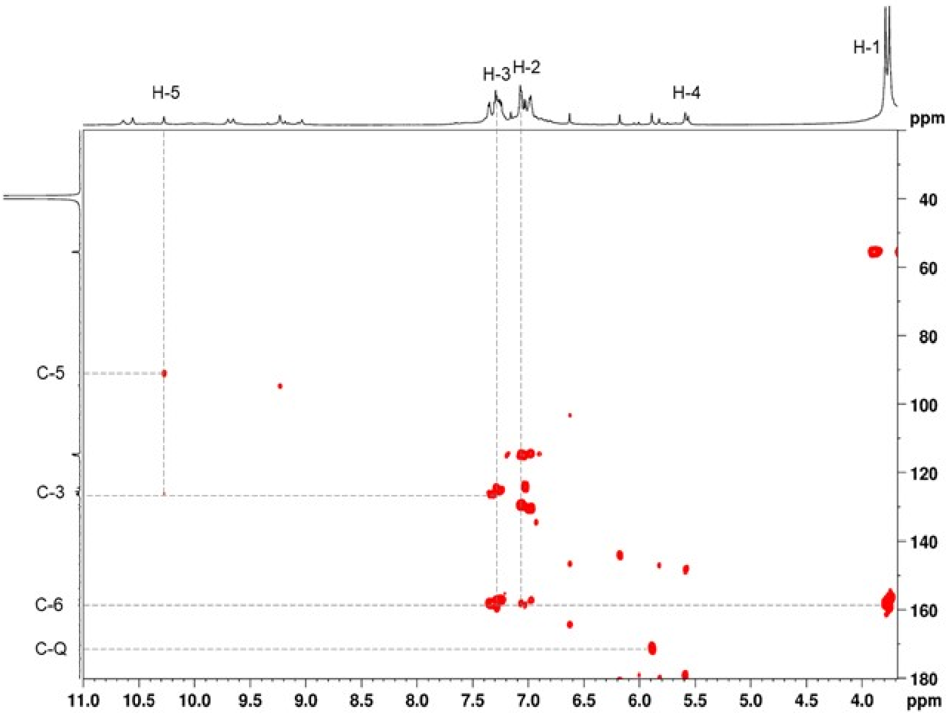

Figure S3. 2D HMBC of PPA in DMSO

assigned with the help of these additional techniques. PPA chemical structure and numerical assignments of carbons and protons are shown in Scheme S1 and are used for the NMR analysis. Figure S2 shows the HSQC NMR spectrum of PPA, where the cross-peaks indicate the correlations between proton chemical shifts and directly bonded carbons. The contour plot displays the direct correlations between the proton signal at δ 3.8 ppm (H-1) with the carbon at δ 55.3 ppm (C-1), the proton signal at δ 7.1 ppm (H-2) with the carbon at 114.5 ppm (C-2), and the proton signal at δ 7.3 ppm (H-3) with the carbon at δ 125.2 ppm (C-3). Rodrigues de Oliveira et al. observed carbon signals at δ 94.9 ppm and 102.0 ppm and assigned them to the isolated carbon (C-4) between the functional groups of the molecular structure.[1] Therefore, the carbon signal observed at δ 94.2 ppm was attributed to the isolated carbon (C-4) in the aromatic structure, and the proton signal at δ 5.6 ppm was identified as H-4.

Figure S3 depicts the HMBC NMR spectrum of PPA, which is suitable for determining long-range ^{1}H-^{13}C connectivity over two or three bonds. In the HMBC spectrum, the cross-peaks at δ 158.3 ppm show an interaction between H-1, H-2, and H-3 and a single carbon signal, which was assigned to the carbon in the aromatic ring that bonds with the methoxy (C-6). The proton signal at δ 10.3 ppm, which does not show any correlation in the HSQC but presents multiple-bond correlations with two carbon signals at δ 90.8 ppm and δ 125.2 ppm (C-3), was attributed to the N-H proton in the polymeric structure. The absence of any other observable correlations between protons nearby N-H with C-6 has led us to propose a polymerization mechanism with the amine group of a monomer bonding to the ortho-position of the amine of an adjacent unit. The HMBC spectrum also shows cross-peaks between δ 171.6 ppm and 179.9 ppm, which have been assigned to -N= bonds from quinoid groups within the polymer structure, as noted by Rodrigues de Oliveira et al.[1] Scheme S1 shows the PPA molecular structure inferred from the NMR data with the polymer consisting of *n* benzenoid repeat units and *m* quinoid repeat units.

Photochronoamperometry (PCA)

UV-deactivated PSI

To validate PSI's contribution to the photoactivity of the composite films, the protein was irradiated with ultraviolet light for 24 h. UV light causes protein photobleaching, and during a full day of UV exposure, yields a predominately deactivated PSI (PSI_d). Figure S4 shows the effect

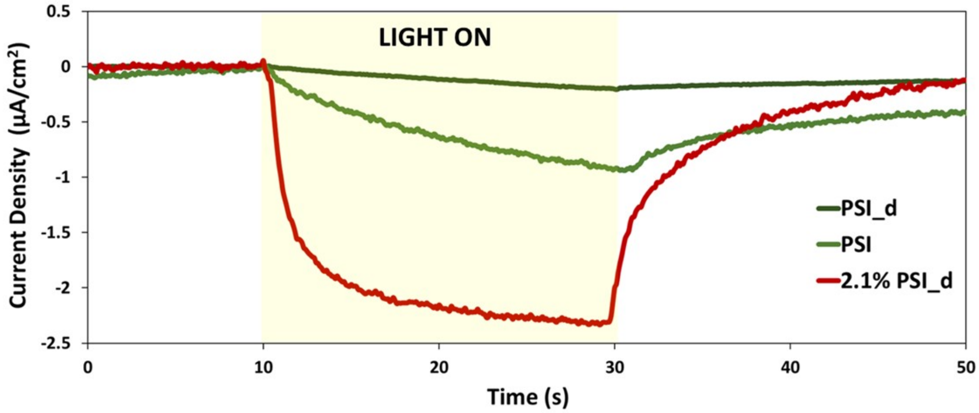

Figure S4. PCA measurements of PSI, partially deactivated PSI (PSI_d), and 2.1% PSI_d in 1 mM redox mediator.

of PSI within a 2.1% PSI film interfaced with the redox mediator solution of 1 mM, and compares the photocurrent of pristine PSI to the photobleached and partially deactivated protein. The UV deactivation caused the peak photocurrent of PSI to decrease by ~4x. Incorporating PSI_d in the 2.1% PSI composite film also yielded a similar decrease in photocurrent, by ~3 x in this case, as compared to the 2.1% PSI composite film in Fig. 7a of the manuscript. These results thereby confirm the importance of active PSI within the composite film.

Photocurrent-Time Rate Analysis

The time required for the photocurrent to reach its maximum value (i_{max}) in Figure 7a depended on the loading of PSI in the composite film. An i-t rate model was adopted to examine the effect

of PSI loading on the rate of photocurrent generation. For this rate analysis, a dimensionless variable $\theta(t) = \frac{i(t)}{i_{max}}$ was introduced in a first order differential equation

$$\frac{d\,\theta}{dt} = a\,(1 - \theta) \qquad \text{(Eq. S1)}$$

where a is a fitting constant with units [s^{-1}]. Confining time evolution from t = 0 s to t = 30 s and setting an initial condition of $\theta = 0$ at t = 10 s, the differential equation becomes

$$\theta = 1 - e^{a(t-10)} \qquad \text{(Eq. S2)}$$

Figure S5a is a plot of the normalized current vs time for the data represented in Figure 7a. With illumination occurring between t=10 to 30 s, fitting θ vs time data to Eq.S2 yields the |a| constant values reported in Figure S5b. While all a values are negative, the larger the |a| value, the quicker θ approaches its maximum value of 1. Pristine PSI and PPA films exhibit the slowest "kinetics" in reaching i_{max}, whereas the composite films reveal a positive correlation between |a| and PSI

a)

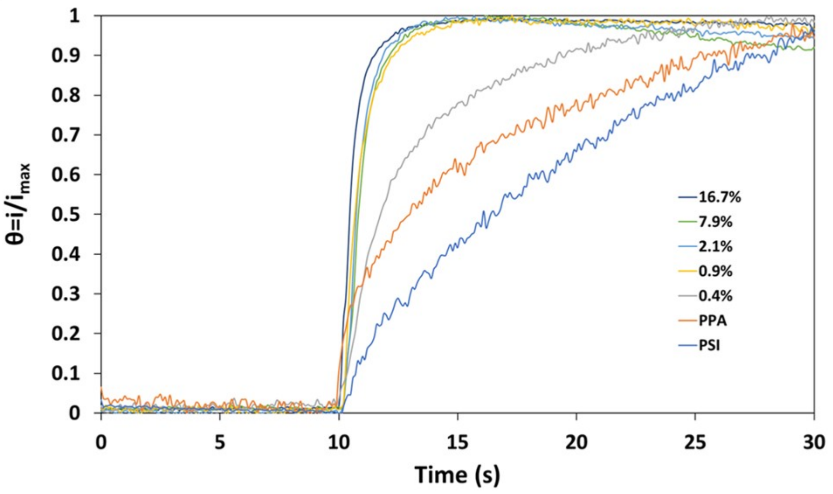

b)

Film	|a| (s^{-1})
PSI	**0.114** ± 0.002
PPA	**0.18** ± 0.02
0.4%	**0.31** ± 0.01
0.9%	**0.99** ± 0.03
2.1%	**1.02** ± 0.05
7.9%	**0.92** ± 0.06
16.7%	**1.54** ± 0.04

Figure S5. a) Normalized current for pristine PPA, pristine PSI, and composite PSI-PPA films at varying %wt PSI, all measured in 1 mM redox mediator concentration with light illumination from t = 10 s to t= 30 s, b) Fitted values of the constant |a| based on Eq. S2.

loading within the film. At PSI percentages at or exceeding 0.9 %, the film reaches its maximum photocurrent in much shorter time span. We attribute this rapid response to the greater absorbance of light by films with a critical loading of PSI, leading to an increased concentration of charge carriers in the film. Nonetheless, even though more light is absorbed at higher PSI loadings, the interplay between light absorbance and conductivity remains in effect, causing the more dilute 2.1% PSI film to exhibit the highest reported photocurrent of these films.

Redox Mediator Concentration

The concentration of the redox mediator couple (potassium hexacyanoferrate(II) trihydrate/potassium hexacyanoferrate(III)) has a dramatic effect on the photocurrent produced by PSI, PPA, and composite PSI-PPA films. Figure S6a is similar to PCA measurements reported in the manuscript with the sole distinction of the films being interfaced with a redox mediator that is at 0.1 mM in solution, which is 10 times less concentrated than the mediator interfaced with the films shown in Figure 7a. At this lower redox mediator concentration, the 7.9% PSI film exhibits the highest photocurrent, in contrast to the 2.1% PSI film exhibiting the highest photocurrent at the higher 1.0 mM concentration of redox mediator. This distinction in peak photocurrent emphasizes the interplay between the redox mediator concentration and the inter-film conductivity and porosity. Figure S6b, which is a compilation of all the peak photocurrent data measured at different %PSI for all composite films, shows that the photocurrent density is higher at 1.0 mM $K_4[Fe(CN)_6]\cdot 3H_2O/K_3[Fe(CN)_6]$ than at 0.1 mM. The higher photocurrents resulting from higher mediator concentrations show that the photocurrents are not limited by

the conductivity of the PPA nor the photoexcitation of the PSI, but by the availability of the

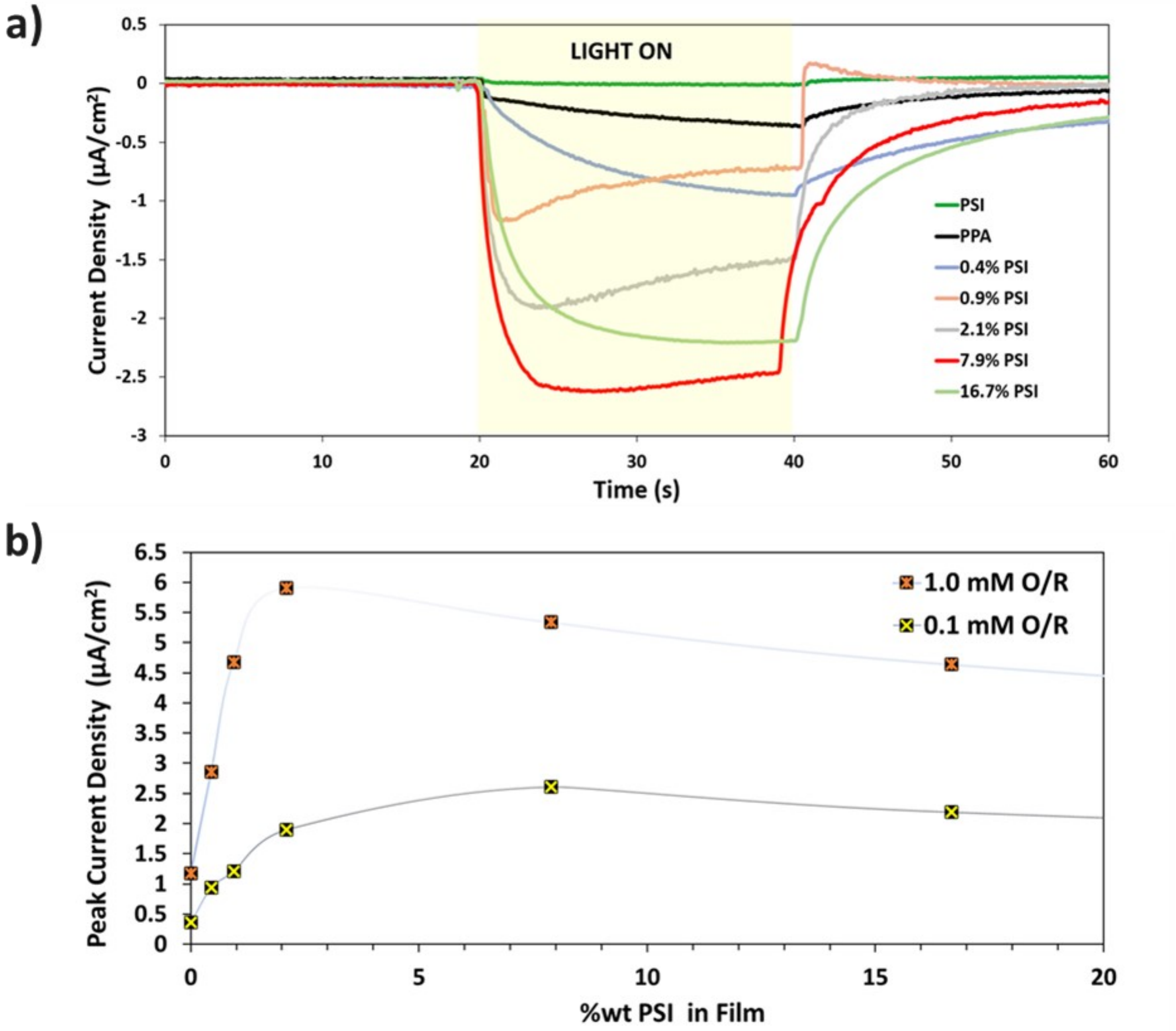

Figure S6. a) Photochronoamperometric measurements of pristine PPA, pristine PSI, and composite PSI-PPA films at varying %wt PSI, all measured in 0.1 mM $K_4[Fe(CN)_6]\cdot 3H_2O/K_3[Fe(CN)_6]$ redox mediator. b) Peak photocurrent densities comparing the photo-response of PPA (0 wt% PSI) and composite films for 0.1 mM and 1.0 mM $K_4[Fe(CN)_6]\cdot 3H_2O/K_3[Fe(CN)_6]$ redox mediator.

mediator.

Reference

1 L. R. de Oliveira, D. de Souza Gonçalves, A. de Souza Carolino, W. M. Facchinatto, D. de Carvalho Menezes, C. O. Dias, L. A. Colnago, Y. L. Ruiz, Ş. Ţălu, H. D. da Fonseca Filho, P. Chaudhuri, P. H. Campelo, Y. P. Mascarenhas and E. A. Sanches, *Molecules*, 2022, **27**, 6326.
